# Epigenetic Clock: DNA Methylation in Aging

**DOI:** 10.1155/2020/1047896

**Published:** 2020-07-08

**Authors:** Shuang Jiang, Yuchen Guo

**Affiliations:** State Key Laboratory of Oral Diseases, National Clinical Research Center for Oral Diseases, West China Hospital of Stomatology, Sichuan University, Chengdu 610041, China

## Abstract

Aging, which is accompanied by decreased organ function and increased disease incidence, limits human lifespan and has attracted investigators for thousands of years. In recent decades, with the rapid development of biology, scientists have shown that epigenetic modifications, especially DNA methylation, are key regulators involved in this process. Regular fluctuations in global DNA methylation levels have been shown to accurately estimate biological age and disease prognosis. In this review, we discuss recent findings regarding the relationship between variations in DNA methylation level patterns and aging. In addition, we introduce the known mechanisms by which DNA methylation regulators affect aging and related diseases. As more studies uncover the mechanisms by which DNA methylation regulates aging, antiaging interventions and treatments for related diseases may be developed that enable human life extension.

## 1. Introduction

Aging is an inevitable biological progress in which the functions of multiple organs gradually decline with time, leading to an increased susceptibility to diseases and environmental stressors [1, 2]. In humans, age has become the primary risk factor for various diseases, such as cardiovascular diseases, cancer, neurodegeneration diseases, and diabetes [3]. Scientists have made unremitting efforts to elucidate the causal mechanisms involved in the phenotypic alterations associated with age. Since many genes appear to show altered expression during the aging process, researchers have focused on studying the long-term effects of environment stress on gene expression regulation. Importantly, epigenetic modifications are proposed to play a crucial role in the progression of aging.

Epigenetic changes are known as genetic variations triggered by the environment and include alterations in histone modifications, DNA methylation, noncoding RNAs, transcription factor binding, and nucleosome positioning [4, 5]. DNA methylation is a biological process by which methyl groups are added to DNA molecules. Histone modifications include lysine methylation, arginine methylation, lysine acetylation, and serine phosphorylation. These modifications alter the extent to which DNA is wrapped around histones and the availability of genes within the DNA to be activated. Recently, numerous studies have emphasized the unique role of DNA methylation in aging [6, 7]. In addition to its effects on the aging process, a function shared by other epigenomic regulators, DNA methylation can also predict aging status. In this review, we will discuss the relationship between DNA methylation and aging as well as summarizing the primary mechanisms by which DNA methylation regulates the aging process.

## 2. DNA Methylation

**Figure 1:**
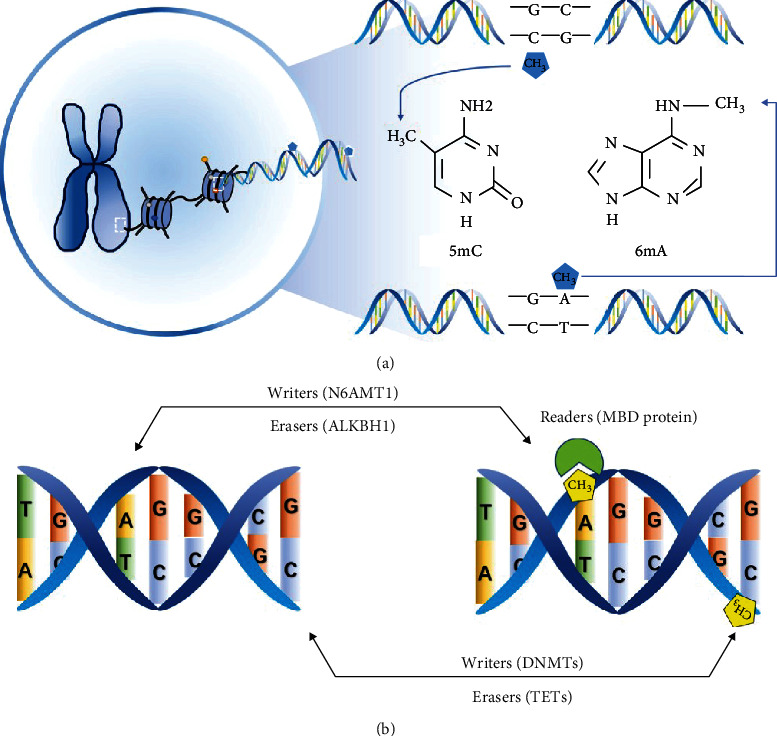
Modulation of DNA methylation in mammals. (a) DNA 5mC methylated site is the fifth position of cytosine, while 6mA modification occurs at the sixth position of adenine in DNA. (b) Methylation marks are established by writers, such as DNMTs and N6AMT1. These modifications are identified by readers, like methyl-CpG-binding domain (MBD) proteins. Erasers, among which TETs and ALKBH1 are representatives, can make all the marks invalid by oxidizing or removing methyl groups.

As is shown in Figure 1, DNA methylation modification involves two different processes: the addition and removal of a methyl group at the fifth position of cytosine or the sixth position of adenine in DNA [8]. DNA 5-methylcytosine (5mC) is the most prevalent DNA methylation modification in eukaryotic genomes and primarily occurs on cytosines that precede a guanine nucleotide (CpG sites) [9]. The presence of 5mC is generally believed to prevent transcription factors from binding to a promoter region and thus suppress gene expression. DNA 6-adenine methylation (6mA) is a recently discovered epigenetic modification in the human genome that has been demonstrated to affect mammalian development [10, 11].

The two types of DNA methylation modifications, 5mC and 6mA, involve different enzymatic systems in mammals (see Table 1). 5mC methylation marks are established by DNA methyltransferases (DNMTs), including DNMT1, DNMT3A, and DNMT3B. DNMT1 is primarily responsible for the maintenance of methylation status across mitosis, while DNMT3A and DNMT3B work in a de novo methylation process. 5mC methylation can be oxidized and changes into 5-hydroxymethylcytosine (5hmC) by ten-eleven translocation (TET) enzymes, which contain TET1, TET2, and TET3 [12]. Moreover, recent studies have revealed the potential role of DNMTs in active DNA demethylation [13]. To date, only a few enzymes involved in 6mA methylation, such as N6AMT1 and ALKBH1, have been identified in mammals [10, 14]. However, a recent study challenged currently used 6mA detection techniques and suggested that more powerful evidence is needed to support the presence of 6mA [15]. DNA methylation modifications typically occur in promoter regions and then recruit classical readers of DNA methylation, methyl-CpG-binding proteins (MBDs). The variation in the expression of DNA methylation-associated enzymes has been observed in some age-related condition, which will be described in greater detail later.

Although continual changes in methylation status are supposed to be adaptive to aging, sometimes this process fails and becomes maladaptive. Ineffective adaptation accelerates the biological aging process and conversely impacts functional and phenotypic aging. Thus, fluctuations in DNA methylation levels in the human genome can serve as an “epigenetic clock” that regularly changes with age [16–18]. Studies have shown that DNA methylation may function as an accurate biomarker to estimate “biological age,” which predicts age-related changes [19]. Furthermore, scientists have begun to understand the potential role of genome-wide patterns of DNA methylation in age-related diseases. To understand the causal connection between DNA methylation and aging, rigorous studies on the underlying biological mechanisms of DNA methylation-induced aging are currently in progress. The mechanisms of aging are complicated and involve multiple factors. Compared with other hallmarks of aging, phenotypic changes resulting from DNA methylation are thought to be associated with a loss of proteostasis, mitochondrial dysfunction, stem cell exhaustion, and immunosenescence [20–22]. Thus, understanding the molecular nature of aging is a key to allow for the identification of targets for interventions to prevent age-related multimorbidity and disability.

## 3. DNA Methylation in Aging

### 3.1. DNA Methylation and Biological Age

It is generally acknowledged that chronological age is not an accurate indicator for the aging process, making it difficult to identify and validate effective measures to promote longevity and healthy aging. Thus, the concept of biological age has been proposed to accurately predict the aging status of an organ or person [23]. Enormous progress has been made in recent years in the development of several potential biological age estimators, of which DNA methylation level is the most promising [19]. Previous studies have clearly proven that genome-wide DNA methylation levels are associated with chronological age throughout the entire human lifespan [24, 25]. Some age-related DNA methylation changes occur in specific regions of the genome and are directional, indicating the existence of differentially methylated regions associated with aging [26]. Therefore, DNA methylation-based biomarkers enable accurate age estimation, which has also been proven by many investigations involving tissues, individuals, and populations [27, 28].

DNA methylation clocks, built from epigenetic DNA methylation marks, rely on the combined use of a mathematical algorithm and sets of CpGs to estimate the biological age of cells, organs, or individuals. In general, age-associated variations in DNA methylation levels comprise locus-specific hypermethylation and global hypomethylation [29]. Since the site-specific CpG methylation fraction can be different in cells from different DNA sources, it has been used to reflect both chronological and biological age.

To date, several human DNA methylation biological clocks have been built that are based on sets of CpGs across tissues and age spectra [30–32]. Analyzing the whole blood samples of 656 individuals, Hannum et al. measured more than 450,000 CpG markers and used the resulting data to generate a quantitative model of aging [30]. In this model, 15% of the identified CpG markers demonstrated a remarkable association between percent DNA methylation and age. Similarly, Horvath built a multitissue predictor of age using 82 published DNA methylation datasets [31]. Interestingly, Horvath also showed that the estimated DNA methylation age of embryonic stem cells was nearly zero and increased with cell passaging, indicating that DNA methylation age is consistent with biological age at the cellular level [31].

Furthermore, the results of a growing body of epidemiological studies indicate remarkably high correlations between DNA methylation-based age and various aging-associated conditions [8]. Detailed assessments of DNA methylation clocks promote a better understanding of the biological foundation of aging and inform us about age-related disease risks. This approach is a typical example of interactions between epigenetics and aging, and its development will be informative and enable subsequent functional studies in humans. Most likely, the most exciting feature of epigenetic clocks is the reversibility of the DNA methylation process, which makes it possible to develop antiaging interventions [33, 34].

### 3.2. DNA Methylation and Age-Related Diseases

Longevity can indeed be regarded as a manifestation of healthy aging. In contrast, accelerated aging is always accompanied by the onset of chronic diseases, particularly degenerative diseases, which ultimately result in disability or premature death. Some researchers also consider age-related degenerative diseases as one of the factors that accelerate the aging process [35]. Emerging evidence indicates that DNA methylation is a crucial factor in age-related diseases.

Cardiovascular disease (CVD), one of the most common age-related diseases, accounted for 31% of global deaths in 2015 [36]. Systematic studies have associated CVD with DNA methylation [37]. In a 10-year follow-up study of 832 participants, increases in biological age, estimated by DNA methylation biomarkers, were accompanied by a 4% rising risk of CVD for each year [38]. CVD risk factors, such as smoking, also induce dysregulated DNA methylation [39]. In contrast, DNA methylation can directly regulate cardiovascular function by modifying the promoters of specific genes and reducing their expression. For example, reduced methylation of the angiotensin I-converting enzyme-encoding gene promoter affects the expression of this gene expression and eventually leads to hypertension [40]. Interestingly, a new role for DNA methylation in the metabolic reprogramming of ischemic cardiomyopathy has recently been discovered, a mechanism that is believed to contribute to the reprogramming of cardiac tissue during ischemia [41].

The incidence of most cancers exponentially increases with age. Aberrant DNA methylation patterns have been observed in variety of tumor cells, including colon, ovarian, and breast cancer cells [42–44], indicating that they may serve as biomarkers in early diagnosis and treatment of cancer [45]. Two DNA methylation modifications appear to be primarily associated with cancer, including the hypomethylation of open sea regions and hypermethylation of promoter CpG islands, with other constituents only playing supplementary roles in the promoter or open sea region methylation process [46, 47]. Current DNA methylation-based biomarker studies focus on the influence of promoter hypermethylation in tumor suppressor genes (such as *RYR2*), which may alter cancer signaling transduction and promote the formation and development of cancer [48–50]. These findings are useful in the development of demethylation drugs aimed at specific tumor treatments.

DNA alterations that occur with age have also been investigated in neurodegenerative diseases. A similar aberrant DNA methylation pattern is shared in patients with Parkinson's disease, Alzheimer's disease, and Down syndrome [51]. During the course of these neurodegenerative diseases, disrupted CpG methylation has been reported to be similar in a set of genes involved in many cellular pathways [51]. Of the identified genes, DNA methylation of the ankyrin 1 gene shows specificity in different brain regions and neurodegenerative diseases [52]. 5hmC has recently been shown to be a potential epigenetic marker in cognitive deterioration [53]. However, additional evidence is needed to demonstrate causality between DNA methylation variability and neurodegenerative disease pathology.

In accordance with these findings, it should be emphasized that DNA methylation has been identified as an early detection maker of age-associated diseases and may also serve as a novel therapeutic target. In vivo studies with animal disease models will be necessary to provide causal evidence of the association between age-related diseases and the aging process.

## 4. Mechanisms of Aging Induced by DNA Methylation

During aging, predefined genes constantly undergo epigenetic modifications and exhibit altered expression in response to internal and external environmental stress. Changes in DNA methylation may occur hundreds of times over the lifespan of an individual in the form of a fully adaptive response. However, in some cases, this methylation acts as a switch for the acceleration of pathological aging, resulting in negative consequences [54]. Thus, global fluctuations in DNA methylation are not only a consequence but also a cause of aging. Understanding the biological mechanisms underlying the observed associations may reveal novel targets for reversing aging-related phenotypes and ultimately prolonging lifespan.

### 4.1. Loss of Proteostasis

Proteostasis maintenance depends on chaperones and two proteolytic systems, the lysosome-autophagy and ubiquitin-proteasome systems [55]. An increase in the disruption of protein homeostasis is one of the primary features of aging. In most organisms, a gradual loss of proteostasis has been observed during aging, and it is reported that long-lived species tend to have more stable proteomes [56]. Additionally, the accumulation of unfolded, misfolded, or aggregated proteins is the leading cause of some neurodegenerative diseases [57].

Evidence has emerged showing that decreased autophagic activity is involved in DNA methylation. DNA methylation inhibits autophagy processes in two ways, one of which is the direct modification and silencing of autophagy-related genes by DNMTs. The promoter regions of *Atg5* and *LC3* are hypermethylated in aged mice, which suppresses gene expression and disrupts the completion of autophagosomes [58]. Whole-body overexpression of *Atg5* results in antiaging phenotypes, extending the median lifespan of mice by 17.2% [59]. Furthermore, researchers have recently shown that DNA methylation inhibitors can rescue phenotypic changes associated with aging by reactivating autophagy-related genes [60, 61].

Another mechanism associated with the dysfunctional autophagy caused by DNA methylation is the modification of genes such as *miR-129-5P* and *FoxO3a*, encoding autophagy-related signaling molecules. In disc degeneration, hypermethylation in the promoter region of *miR-129-5P* reduces gene expression, which then blocks autophagy through downregulation of *Beclin-1* [62]. *FoxO3a* is a protective transcriptional regulator that maintains cell homeostasis from environmental stress by increasing autophagy [63]. DNA hypermethylation of the *FoxO3a* promoter indirectly inhibits autophagy, contributing to aging-related endothelial dysfunction [64]. Abnormal methylation in some tumor-related genes, such as *TCF21*, *CELF2*, and *NOR1*, is also involved in autophagy regulation, which contributes to some premature aging disorders [65–67].

While DNA methylation has gained recognition for its involvement in protein degradation, information regarding its effect on protein synthesis in senescent cells is just beginning to emerge. The expression of ribosomal RNA (rRNA) determines translational rate and protein synthesis, which decreases during physiological aging. It has been reported that abnormal methylation of ribosomal DNA (rDNA) promoters occurs with age [68]. Recent studies have confirmed that increased CpG methylation in rDNA promoter regions inhibits transcription and thus significantly reduces the expression of 18S, 5.8S, and 28S rRNA [69]. Furthermore, DNMTs have been shown to affect the synthesis of proteins associated with long-term memory, providing an explanation for memory impairments that occur with age [20].

Speculation has increased that experimental perturbation of proteostasis precipitates pathological change and accelerates aging. Therefore, further studies are needed to determine if genetic manipulations can successfully maintain proteostasis in aged mammals.

### 4.2. Mitochondrial Dysfunction

Mitochondria are considered to be the powerhouses of cells and are important for energy production through respiration and cellular metabolism regulation [70]. The accumulation of damage to mitochondria can reduce energy metabolism and increase the production of reactive oxidative species (ROS), leading to aging-associated changes. The expression of mitochondrial methyltransferases has been shown to be age-dependent, indicating that mitochondria are involved in the development of proaging features [71].

Increasing methylation of *Elovl2* has recently been reported to be a crucial driver of aging by inducing a stress response in the endoplasmic reticulum and promoting mitochondrial dysfunction [6]. In some degenerative diseases, mitochondrial DNA and genes encoding the enzymes responsible for mitochondrial biogenesis, such as *Mfn2* in diabetes, are hypermethylated and compromise the electron transport chain [72]. Increasing methylation of the D-loop region and mitochondrial NADH dehydrogenase 6 causes insulin resistance in obese individuals, which can easily develop during aging [73]. The upregulation of 5mC and DNMTs in neuronal mitochondria has been postulated to be an important feature of neurodegeneration. In addition, DNMT inhibitors can improve the protection against oxidative damage in senescent cells [74]. There is also a positive correlation between DNA methylation and base mismatch in the dysfunctional mitochondria of vascular diseases [75]. Moreover, the DNMT inhibitor 5-azacytidine has recently been shown to reverse the aged phenotype of mesenchymal stem cells (MSCs) by reducing ROS and nitric oxide levels, the accumulation of which results in low viability and mitochondrial dysfunction [21].

These findings raise the possibility that rejuvenation of mitochondrial dysfunction may be a potential approach for prolonging life. Thus, further investigation is warranted with respect to determining whether directly targeting DNA methylation can ameliorate mitochondrial damage and delay aging progression using animal models.

### 4.3. Stem Cell Exhaustion

Adult stem cells are of great importance in maintaining tissue homeostasis and regeneration over a lifetime. Stem cell exhaustion can be described as a qualitative and quantitative decline of stem cells. This process has been observed in many senescent tissues and organs and is regarded as one of the driving forces of aging. Importantly, epigenetic regulators have been shown to control stem cell fate [76, 77]. The role of epigenetic dysregulation in stem cell exhaustion has recently become the subject of intense research, and DNA methylation is thought to be an age-dependent upstream regulatory factor affecting cell-specific gene expression as stem cells become more specialized.

A great deal of evidence has revealed that changes of DNA methylation level regulate genes involved in self-renewal in aging hematopoietic stem cells (HSCs). Adelman et al. demonstrated that epigenetic reprogramming of human HSCs, including redistribution of DNA methylation, occurs with age [78]. Supporting this finding, the inactivation of DNMT3a enhances cell self-renewal at the cost of differentiation potential in vivo [79]. Ablation of both DNMT3a and DNMT3b results in a more severe effect on cell differentiation capacity, which is one of the characteristics of natural HSC aging [80]. A deficiency of DNA demethylases, known as TET, yields similar results, which reduces genomic levels of 5hmC and contributes to lineage skewing towards myelopoiesis in HSCs [81]. Further evidence has revealed a direct connection between TET and HSC function in leukemogenesis [82].

Significant differences in DNA methylation levels are observed at specific CpG sites, especially in differentiation-related genes, throughout both the aging processes and long-term cultures of MSCs [83]. The DNMT inhibitor RG108 has been reported to modulate the transcription of prosenescence genes and alleviate oxidative stress-mediated damage in human bone marrow-derived MSCs (BM-MSCs) [74, 84]. DNMT3a and DNMT3b methylate the promoters of stem cell functional genes during the chondrogenic differentiation of BM-MSCs, and the reversion process can be regulated by the demethylating agent 5-azacitidine [85]. Similar results have been obtained in human hair follicle MSCs in which the upregulation of DNMTs suppresses downstream genes associated with stem cell properties, which is essential for maintaining self-renewal capacity and reversing cell senescence [86]. In addition, the DNA 6mA demethylase ALKBH1 has also been shown to affect the ability of MSCs to differentiate, providing new evidence of the potential regulatory role of DNA 6mA in mediating the aging process [87].

Considering the gradual decline of regenerative ability in muscle tissue with age, muscle stem cell (MuSC) senescence is of great concern. There are many studies on the relationship between age-related alterations of DNA methylation and transcriptional variability in senescent MuSCs. Changes in DNMT and TET expression have been observed in MuSCs resulting from proliferation to differentiation and between quiescence and proliferation [88, 89]. Furthermore, the regeneration of skeletal muscle after injury is markedly inhibited in *Dnmt3a*-knockout mice [90]. Bigot et al. observed that age-related DNA methylation in MuSCs primarily acts on the sprouty1 pathway and that through its suppression, the self-renewal capacity of senescent MuSCs is destroyed [91].

### 4.4. Immunosenescence

The deterioration of the immune system in aging, known as immunosenescence, is characterized by immune lineage skewing and higher levels of inflammatory markers. This process is one of the causes of inflammaging, a sterile, low-grade, and chronic proinflammatory condition of older organisms [22]. Tserel et al. observed different DNA methylation levels and skewing in human CD8+ T cells isolated from different age groups [92]. Age-related DNA methylation involves variations in both the levels of immune-related factors and the proportions of immune cell types.

A recent study observed age-sensitive hypermethylation in the promotor region of *Klf14* in several human tissues, which affects the differentiation of CD4+ T cells via suppression of *FOXP3* [93]. Garg et al. demonstrated that regulatory T cells from aged mice have an intensified inhibitory impact on effector T cells due to the hypomethylation of the *FoxP3* enhancer, which consequently increases immune suppression with age [94]. Similarly, the methylation content of *GSTM1* is involved in type 1 T helper cell differentiation [95]. Alterations in *Tet2* expression have been observed in myeloid malignancies, and TET2 has been shown to regulate myeloid and erythroid lineage differentiation [96]. The deficiency of DNMT1 in mice has been suggested to cause immune senescence and is involved in the development of early autoimmunity [97].

Abnormal DNA methylation induced by aging also accompanies disturbances in inflammatory cytokines. Shinozaki et al. measured DNA methylation levels in brain and blood samples and observed a significant negative association between aging and the DNA methylation of inflammatory factor genes (such as TNF-alpha and IL-6) [98]. Methylation levels in the mouse *Klf14* promoter region increase with age and obesity and appear to be a regulatory factor of chronic inflammation in adipose tissue [99]. Furthermore, elevated 5hmC levels have been shown to promote the appearance of Iba1-positive inflammatory microglia in a study investigating age-related cerebrovascular alterations [100]. DNA methylation has also been suggested to have a role in the development of inflammation in several age-associated chronic diseases, such as cancer, osteoarthritis, and neurodegenerative diseases [101–103].

## 5. Prospects and Conclusion

The underlying mechanisms of aging have perplexed scientists for decades. Detailed evaluations of global DNA methylation changes have provided insights into the process of aging, with DNA methylation serving as a biomarker of biological age and a driving force of aging. Current findings should stimulate further discussion and experimentation based on epigenetic regulation in cell-, tissue-, and disease-specific aging models. Future studies focusing on the mechanisms by which specific parameters, such as stress, affect methylation patterns will uncover additional details of the aging process. Identification of the target genes modified by DNA methylation-related regulatory elements in aging individuals is highly informative to figure out the hormone-like effectors and signal pathways that mediate these alterations as well as related diseases. The interaction among epigenetic regulators during aging should also be highly valued. Further studies should focus on the cross-talk among these epigenetic regulators, such as DNA methylation, RNA methylation, histone methylation, and noncoding RNAs, which will aid in providing a full picture of epigenetics and aging. The results of such studies may pave the way for antiaging interventions as well as treatments for related diseases, enabling human life extension.

## Figures and Tables

**Table 1:** Enzymes involved in mammalian DNA methylation.

Type of DNA methylation	Role of enzymes	Family	Members
5mC	Writer	DNMT	DNMT1
			DNMT3A
			DNMT3B
	Eraser	TET	TET1
			TET2
			TET3
	Reader	MBD	MeCP2
			MBD1
			MBD2

6mA	Writer	HemK	N6AMT1
		Mettl	Mettl4
	Eraser	ALKBH	ALKBH1
			ALKBH4
